# Combating female genital mutilation in Northeast (Horn) Africa and its challenges

**DOI:** 10.4314/ahs.v20i4.54

**Published:** 2020-12

**Authors:** Amirbahram Arabahmadi

**Affiliations:** University of Tehran

**Keywords:** Female genital mutilation, Northeast Africa, international organizations, nongovernmental organizations, gender discrimination

## Abstract

**Background:**

This article investigates the practice of female genital mutilation as a long-held custom in the countries of Northeast Africa, known as Horn of Africa, where many women in rural and urban areas are faced with different physical and psychic consequences in their future lives.

**Objective:**

To investigate the prevalence of FGM in the Horn of Africa and the traditional thinking of People about it.

**Methods:**

This study was based on descriptive analysis method. The questions of the study are (a) Why female circumcision is widely practiced in Horn of Africa; (b) What are the mental and physical consequences of female genital mutilation for the women; and (c) How regional and international entities, whether governmental or NGOs, are combating this tradition.

**Results:**

This article has found out that female genital mutilation in Northeast African countries has resulted in many lifelong diseases and sexual degradation in many women and the best way to combat this tradition is to inform people by gradual (not abrupt) trainings without any insult to the beliefs of the people.

**Conclusion:**

This study reveals the Health education based on behavioral change. In doing so, the unity of policies between regional and international actors along with attracting the support of tribal elites is also needed.

## Introduction

**Figure 1:**
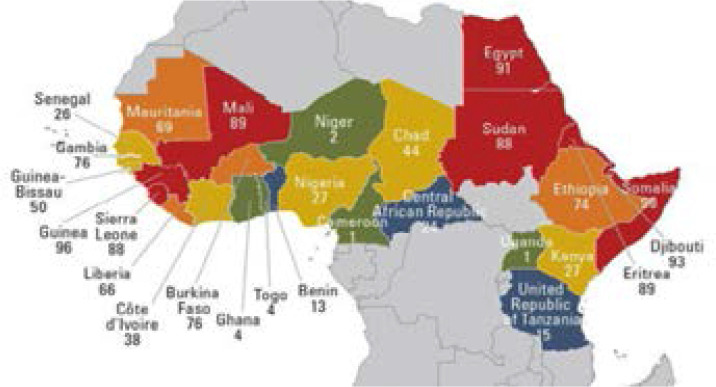
[56]

The African continent contains ancient customs that form a large part of the oral culture and customary laws of the various countries. These traditions, passed down from generation to generation, continue to be held in high esteem among the African tribes, and the People of this vast continent consider the implementation of these traditions as a guarantee of the stability of their oral culture. Although most of the African peoples' traditions have adapted to the realities of the contemporary period, there are still a few traditions that continue to be based on some traditional beliefs and cause irreparable physical and psychological harm to African women. Figure 1 shows percentage of female genital mutilation in different countries of Africa (2016–18) 1

Female genital mutilation is among the many tribal traditions which are still practiced by some African countries. According to the statistics of the World Health Organization, this action is currently performed in thirty African countries 2 with nearly 92 million African women, out of the total number of 200 million women worldwide, who have experienced this practice. Each year, an estimated three million girls are expected to experience genital mutilation 3.

Female genital mutilation, sometimes also called as “female circumcision,” and “female genital cutting” 3 is an act of cutting part of the female genital usually without any kind of sanity, only to observe old ethnic customs of tribes. In Horn of Africa, as well as other regions of Africa, female circumcision is often done in traditional styles, without using sterile and sanitary tools, accompanied with pain and aching. This is done by cutting a part of the girl's genital, using cutting utensils such as knives, scissors, ruler, blade or even a piece of glass and then stitching it up with thread; thereafter, things like oil, honey, yogurt and tree leaves are applied to stop the bleeding. 4 Figure 2 shows Traditional women's circumcision tools in Northeastern Africa - rural areas.

**Figure 25:**
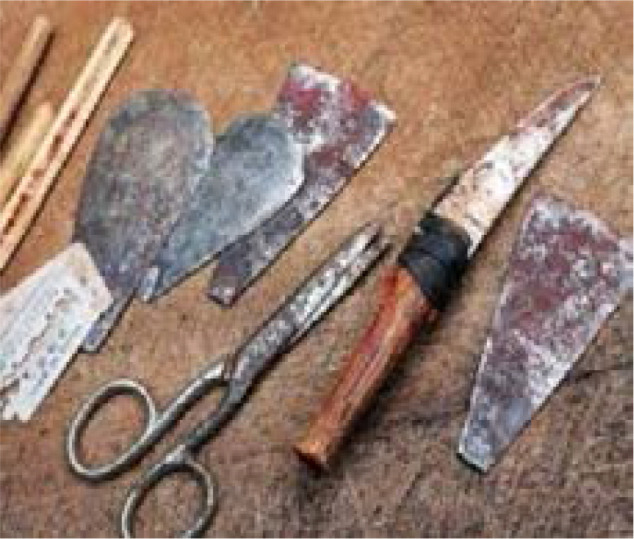


In these countries, female circumcision usually takes place from ages 1 to 9 and 15 to 49 6. It cannot be precisely said that this practice is done only on the women in ages between 15 to 49, yet any girl from her birthday to maturity is potentially in danger of being circumcised^7^. While a number of female genital circumcision is operated with sanitary tools by trained nurses, yet still many other practices are done by traditional old women and tribal midwives or even barbers who know nothing of the medical cares 8.

There is no precise date attributed to the earliest practice of female circumcision in the world. However, according to the famous Greek historian and geographer, Herodotus (425–484 B. C.), female genital mutilation were common among the Egyptians1 at the time of Ancient Egypt 9 as well as other people like Phoenicians, Hittites and Ethiopians who also used to do this practice since old times. However, other nations like Rome empire also used to practice female circumcision; therefore, it was not limited to ancient Egypt or other African countries 10. Female genital mutilation in Africa has also a long history and hundred years before the entrance of Islam or Christianity, FGM occurred in this continent 8. This rite is recognized as a part of the old ethnic tribal traditions of Africa and has been present in Africa for at least two thousand years, and the indigenous population insists on maintaining it and its particular characteristics.

The Horn of Africa, a peninsula in Northeast Africa, is home to the countries of Somalia, Eritrea, Ethiopia, Djibouti (and to some extent Sudan), and according to the WHO's statistics 11 is the location with the highest number of female circumcisions in the world, including in its most severe forms, with more than eighty percent of women in the region -from 15 to 49 ages- experiencing this practice 12. Table 1 indicates estimated prevalence of FGM by age in the Horn of Africa (2018)

**Table 1:** 13

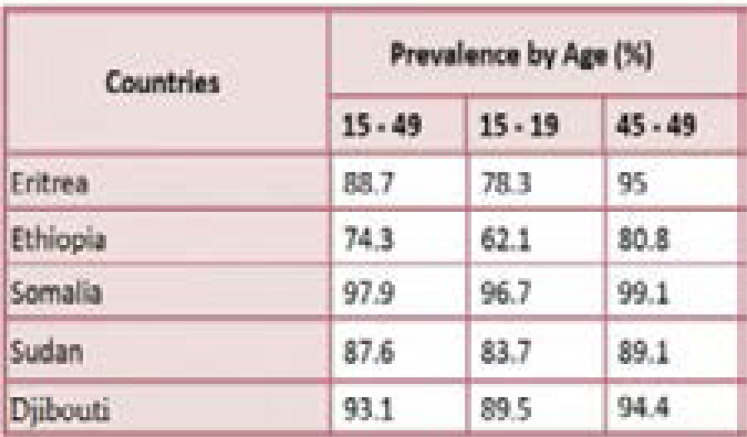

In fact FGM has been part of the recognized tradition of the ethnic groups residing in the Horn of African, with these practices being practiced for at least two thousand years. The people of the Horn of Africa have preserved the tradition in its full manner to the present day. This is the key reason why this region has been chosen as the case study for this research. The table below (Table 2) shows the latest statistics regarding female genital mutilation in 2018 for women aged 15 to 49 in Northeast Africa (although the practice may have taken place at any time from birth until puberty). 14

**Table:** 21

No	country	percentage
1	Ethiopia	74%
2	Eritrea	89%
3	Djibouti	93%
4	Somalia	98%
5	Sudan	88%

This paper tries to investigate historical roots of this simple tradition and the reasons behind its current practice. The main hypothesis is that the persistence of the implementation of old traditions and oral cultures in the Horn of Africa is the main reason behind the continuation of FGM in the region up to the contemporary period. The main research questions addressed here are (a) Why female circumcision is widely practiced in the Horn of Africa; (b) What the mental and physical consequences of female genital mutilation for the women are; and (c) How regional and international entities, whether governmental or NGOs and international organizations, are combating this tradition.

A literature review of this subject reveals that many scholarly articles have been written on female genital mutilation in the Horn of Africa. However, this paper is novel in its attempt to examine the conflict between tradition and modernity in the region in the context of the phenomenon of female circumcision as an old tradition and the duality of the new and older generations in preserving or abandoning it, drawing upon reliable documentation to do so.

## Methods

This paper uses the method of descriptive content analysis. Descriptive content analysis aims to identify and describe the main content of the data, chronologically, thematically or otherwise, either subject-focused or researcher-focused, and involves counting, listing, practicalizing and categorizing, as well as evaluation and interpretation. 15 In this paper the phenomenon of FGM in the Horn of Africa and its consequences such as its profundity among peoples, the reasons behind it, the role of NGOs and international organizations to eradicate it as well as FGM challenges in this area have been scrutinized based on a descriptive content analysis framework. In addition, some of the evidence for this paper such as a friendly conversation with ordinary and intellectuals as well as interviews with some urban ladies was collected by the researcher during several short journeys to the Horn of Africa countries such as Ethiopia, Eritrea, and Sudan (approved by Department of Social Anthropology of Addis Ababa University 2019), and while not directly drawing upon his area studies expertise in this paper, due to his acquaintance with the Horn of Africa countries' culture and traditions, the author has interacted respectfully with the subject of examination.

### Data

The data collected and presented in this paper are divided into two parts. Much of the material was collected from books and credible databases, and the remainder is drawn from the analysis of the author's questionnaire responses from a sample of women from the Horn of Africa.

### Types of Female Genital Mutilation (As defined by WHO)

Generally, there are four types of female genital cutting with subdivisions 3 in countries in Horn of Africa. These include:

- I – Partial or total removal of the clitoris and/or the prepuce (clitoridectomy). When it is important to distinguish between the major variations of Type I mutilation, the following subdivisions are proposed:
  - Type Ia, removal of the clitoral hood or prepuce only;
  - Type Ib, removal of the clitoris with the prepuce.
- Type II – Partial or total removal of the clitoris and the labia minora, with or without excision of the labia majora (excision). When it is important to distinguish between the major variations that have been documented, the following subdivisions are proposed:
  - Type IIa, removal of the labia minora only;
  - Type IIb, partial or total removal of the clitoris and the labia minora;
  - Type IIc, partial or total removal of the clitoris, the labia minora and the labia majora.
- Type III – Narrowing of the vaginal orifice with creation of a covering seal by cutting and appositioning the labia minora and/or the labia majora, with or without excision of the clitoris (infibulation). When it is important to distinguish between variations in infibulations, the following subdivisions are proposed:
  - Type IIIa, removal and apposition of the labia minora;
  - Type IIIb, removal and apposition of the labia majora.
- Type IV – All other harmful procedures to the female genitalia for non-medical purposes, for example: pricking, piercing, incising, scraping and cauterization.^16^ Although all four types are prevalent in Horn of Africa, infibulation is mostly practiced in the Horn of Africa, namely lowland Ethiopia, Djibouti, Somalia 17 and Sudan^14^ where the prevalence is the highest in the world.^18^

## Findings and Discussion

There is no doubt that FGM exists with an extensive variety of temporary and long-standing health risks comprising physical, psychological, sexual, and obstetric health risks 19 that we are going to talk about them briefly. Meanwhile, although the author gained valuable insights during his travels to Eritrea, Sudan, and Ethiopia by interviewing several academics in these countries, he prefers not to refer to the interviewees directly in order to maintain the confidentiality of his sources. Therefore, the bulk of the references given below relate to information collected from published sources.

### Physical complications

After circumcision, women in the countries of the Horn of Africa are faced with harmful mental and physical symptoms including infection, uterus cyst and awful pain during sexual intercourse, childbirth with pain and burden, repetitious urine, consistent bleedings and many other physical and mental problems 2. Other physical implications include pain during menstrual cycles, increasing bleeding risk and infection during pregnancy and hepatitis. These symptoms may have lifetime circumstances for those women who have experienced any of the four types of circumcision 10.

Moreover, circumcision among girls is one of the main reasons for the early death of the women in Horn of Africa, in a way that a huge number of infant or teenage girls who has experienced genital circumcision die because of severe bleeding, infectious disease or relates illnesses 19. On the other hand, due to the short openness of uterus of circumcised women, 10 mortality rate of babies during the time of delivery is very high. 20

That is why almost one out of four persons in the Horn of Africa (and similarly in the whole Africa) with their genital being circumcised, dies due to the infection in their urine and uterus organs. In addition, those women have ongoing pains and severe bleedings during their time of delivery 21.

### Psychological complications

The implications are not limited to physical illness, but are accompanied with severe mental and psychological circumstances 10. Females experiencing physical complications often identify their father as the party responsible for these difficulties. Furthermore, female circumcision also disturbs an element of the sexual relationships of couples 22. There is evidence that women with FGM/C are more likely to experience anxiety, somatization, phobia, and low self-esteem than those without genital mutilation 23.

Circumcised women in the Horn of Africa usually consider this practice as one of their permanent anguishes until their death. This anguish is highly manifested in three phases of their lives; one is at the time of circumcision in childhood, the other is at the time of wedding night, and the third is when they want to give birth to their first child 24.

### Provided Justifications for the Practice:

There are many provided justifications for why still these practices are being practiced in the countries of Horn of Africa. 25 These are mostly interlinked with the existing different ethnic and tribal cultures, family relations, tribal connections, social class, economic and social circumstances, and education. 26 One of these reasons that is mentioned by most of Horn of African families regarding why they are letting their daughters being circumcised is that they think she may not be able to marry a man if she is not circumcised 2. Actually, they are afraid that if their daughters are not circumcised, other may think of them as being without decency and purity. Additionally, there is a common belief that by doing circumcision on young girls, they could properly control their sinful ambitions when they become mature^27^. Moreover, there is an old traditional belief among the people of the Horn of Africa that attributes male characteristics to the female organ of clitoris, believing that “cutting it protects female mortality over them”^28^. Accordingly, in the oral tradition of many tribes in this region, when babies are born, they are bisexual by nature, and it is only after their circumcision that their sexuality is determined.

It is also widely accepted by the people in the Horn of Africa, that whoever have not their genital cut at their childhood, then they may confront with numerous physical problems at the time of their child delivery after marriage, and that does not end here, since they believe that a mother without circumcision could not be able to have deep relationship with her newborn baby. Besides, there is this believe among the African people that circumcised women have better fertility capabilities^8^. Table 3 shows the responses provided to the author's questionnaires.

**Table 3:** Benefits of girls undergoing FGM procedures as identified by educated women in the Horn of Africa^29^

Country	Respect for customary laws	Maintaining tradition	Social dignity	Marriage guarantee	Virginity preservation	Other
Eritrea	15	14	19	31	36	18
Somali	30	25	28	35	39	15
Djibouti	17	18	15	29	33	19
Ethiopia	11	14	16	27	30	17
Sudan	9	11	20	27	36	10

Based on descriptive content analysis as well as qualitative analysis through translation, transcription, validation, and codification of personal information from questionnaires and interviews; valuable information was extracted: Most study participants had some education, fluctuating from high school graduates to university grades. Educations was reported by respondents while most of them had professions as civil servants, health workers, or were involved in trading. Contributors' ages were also varied from 40 to 65. Almost all of them were married and had male and female children, and being involved in the practice of FGM. Participants had been assured that their name will be anonymous and were requested to give trustworthy information. Based on the information author has gathered through direct interviews as well as questionnaires testifies that despite the enlightens by governments and NGOs, there are still a lot of myths and misconceptions about the clitoris among the Horn African women and interviewees ascribed several negative outcomes to uncut women, shows utterly hidden beliefs of female interviewees against uncut women, consider them as a cultural taboo and contrary to their roles as good mothers and wives and even predisposing some of them to marital infidelity. It was interesting for the author to know that most of these negative opinions deriving from narratives founded upon accounts by older people and were not necessarily experiential. Another important issue that was revealed to the author was that decision-maker for whether a girl is cut or not is usually husband and undeniably fathers seemed to hold the power in final decision-making. Therefore, although FGM has been described as a “tradition in transition”^27^ there is still a long way to control it at least in this part of the Africa continnt.

Additionally, according to traditions, people of this region believe that circumcision is a way of an introduction that the girl has entered puberty and is ready to get marry and make a family 27. In the other words, female circumcision is considered as a value at the time of marriage, in a way that if the virginity of the bride could not be identified on the first night of the marriage, the groom's family can bring her back to her father's house. Other than that, many young men are unwilling to marry the uncircumcised girls. Even the amount of Cabin (dowry) for a circumcised bride is more than those who have not been circumcised 30. Health benefits and male sexual enjoyment are also factors in the continuation of the practice, albeit less influential ones.^31^ Figure 3 displays the conceptual model of factors perpetuating FGM in Northeastern Africa.

**Figure 332:**
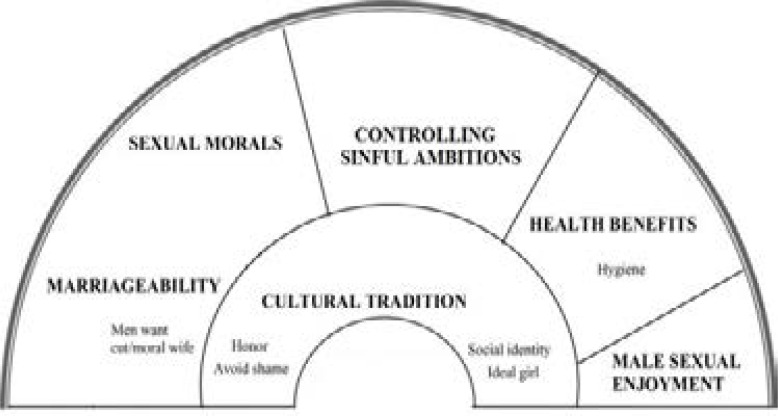


## Limitations

Some parts of the current study are extracted information limited by the participants from different ethnic groups of Horn African countries; therefore, outcomes are not normally appropriate to a precise ethnic group nor do they essentially reproduce the experience of all genitally mutilated women living in the Horn of Africa. Apart from that, FGM is still considered as a secret or cultural and social taboo in African countries and among ladies especially when they want to talk about it with strangers, particularly when they want to express they feeling about it whilst they are still reluctant between tradition and modernity or in the other word in preservation of their customs or let some of them be updated. So in such cases convince them to participate in data analysis through questionnaires is a big task. Clarification of the extracted results during the data analysis to have accurate and confirmed results was another difficult task too. Guaranteeing them that their name and specifications will be completely unidentified was very problematic as well.

## Anti FGM Campaign

### Governments

In the state-level struggle to abolish female circumcision or at least to gradually decrease the practice of this tradition, many states in the Horn of Africa have done many great jobs in this regard. Hence, Ethiopia in 1997^33^, Eritrea in 2007 34 and Djibouti in 1995 and 2009 35, have recognized FGM as an act of crime and those who are encouraging or operating female genital mutilation are considered as criminals. Therefore, in these countries, it is by law now that any case of female genital mutilation will face a sentence of six months to many years of imprisonment along with a payment of a large amount of money 36. Although FGM is yet to be officially prohibited in Sudan; however, one can sue a case of forced female circumcision in courts or tribunals^37^. Sudan, in cooperation with international organizations, has also provided educational curriculum and workshops for elementary schools regarding the dangers of female genital mutilation. Another thing that governments are doing in combating FGM is that local artists are encouraged to make artifacts about the risks of female circumcision and provide local and international institutions with some especial facilities 38.

Other than state governments, the national assemblies of twenty African countries – including those of Horn of African countries – in cooperation with the United Nations and the African Union Parliament, are also trying to encourage their own people to fight female genital mutilation by expanding the educational activities. Over the last few years, many of the speakers of these national assemblies have recognized that some of the ancient ethnic tribal traditions of the African continent, including female circumcision, must thoroughly be revised. They emphasize that some of the oral cultures are not compatible with divine religions 38. During a conference in Senegal in 2010, African National Assemblies formally asked the UN General Assembly to consider Female Genital Mutilation as an illegitimate act in all countries of the World, since all the good efforts being done to combat this practice within the African countries in the last few years 6.

These efforts were not limited to governments, as local groups and ethnic societies have also performed a great job in making native people aware of dangerous consequences of female genital mutilation for girls and women, and eventually could convince many of those people of not doing so. These local groups or societies, whether in small or rather big communities, are extremely committed to sayings of their tribal leaders. In fact in Horn of Africa, many of these efforts - done by international organizations, NGOs or state governments - to combat female circumcision were done through persuading the chiefs of tribes; the result of which was that some local groups have formally accepted to end female circumcision within their tribes 39. Since 1988, the Djibouti Women's Association has run a huge campaign to ban this tradition 40 In addition, after a religious Fatwa in Somaliland, the government of this self-declared state in Somalia formally considered female circumcision as a sinful act in 2018 41.

Because of these efforts in recent decade, nearly 2000 local groups all over Africa, including those in Horn of Africa, have announced their disapproval of female circumcision 36.

### NGOs

In recent decades and especially since the early 1980s, along with the international organizations, NGOs as their most important priority, have been trying to fight female genital mutilation in the countries of the Horn of Africa. The first non-governmental organizations started combating female genital circumcision in Ethiopia and then it overspread to other countries of the region including Sudan, Ethiopia, Djibouti and Eritrea^42^. One of the main activities of these nongovernmental organizations is to improve the general knowledge of the people about the consequences and dangerous of the female circumcision. The procedure of educating urban and rural people in the Horn of Africa regarding the disadvantages of female circumcision is done via different mass communication tools including publishing leaflets and posters, holding public gatherings and lectures at schools; however, trying to change the popular culture of this area needs more time 36. Figure 4 shows a billboard in the Horn of Africa on the consequences of Women's Genital Mutilation.

**Figure 441:**
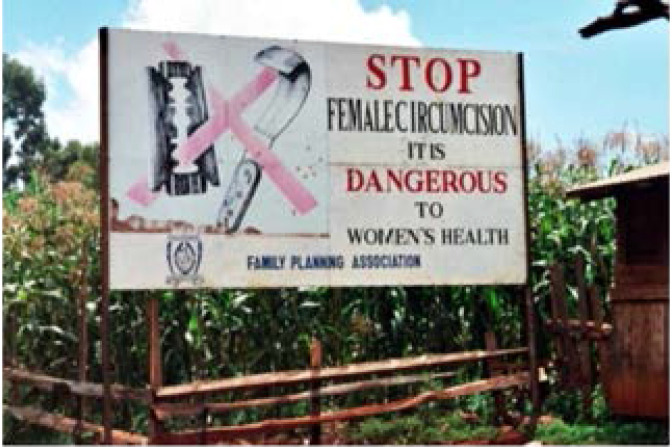


As it is evident, apart from other aspects; a significant part of “Anti FGM Campaign” is based on education and educational programs. That's why the Governments, NGOs and international and regional organizations use mass media (especially radio and television networks), Billboards and posters, public meeting and lectures in Schools and Universities to increase public awareness of the dangers and harms of Female Circumcision.^44^ Also, since education plays an important role in reducing the rate of FGM in Horn of Africa, it has been found that educated parents who also care about their daughter's education, are less likely to practice FGM. For this very reason, Governments and non-governmental organizations in the Horn of Africa have made extensive effort to familiarize girls with their individual and social rights by adding such topic to the schoolurricula.^45^

### Regional organizations

Alongside the NGOs, the African Union which is the largest regional organization consisting of all states of the African continent, brought the topic of female genital mutilation into its agenda as early as its foundation. A year after that in 2003, the African Union issued a protocol named the Maputo Protocol that protects the rights of girls and women against circumcision. In 2004, this Protocol was declared to all member states of the African Union; and countries of the Horn of Africa have either ratified it or are trying to ratify it in their national assemblies. Table 4 shows legislations against FGM in Horn African countries.

**Table 4:** 46

States	Year
Sudan(Some states)	2008–2009
Ethiopia	2004
Djibouti	2009
Eritrea	2007
Somalia	2012

It is expected by the African Union that the phenomenon of FGM in the Horn African region be eliminated by 2030, so that the next generation of African girls would be protected from this hazardous action 47. Dozens of seminars and training workshop are held by the African Union in many countries of the Horn of Africa that so far could reduce the total number of female genital mutilation. Beside the people, other local organizations could also get benefit of the guidelines of the African Union in terms of the disadvantages of the female circumcision.

## Challenges and Difficulties

Despite all international and regional efforts and campaigns against female genital mutilation in Africa, still in the Horn of Africa there are several challenges and difficulties for the total extermination of female circumcision, partly because of the region's ethnic and tribal rituals. These obstacles and challenges include - but not limited to - the lack of literacy or schooling, firm convictions and social prejudices, and pledge to the long-standing customs and rituals among the people. Actually, people (especially villagers) of the Horn of Africa even refrain from talking about the female genital mutilation, for it is viewed as socially unmentionable or taboo 48.

Besides, while there is legal ban on female circumcision in countries of Horn of Africa, yet there is no executive body for exercising this law or any punishment for disobeying the law 49. Actually, in Horn of Africa, those individuals and organization that are against female genital mutilation are faced with the harsh sarcasm of other people (especially other women) in the tribe who label them as anti-traditional, anti-religious, and anti-cultural 19. In addition, while in some African countries, the risks of circumcision for the health of women are taught in schools, yet some traditional families, who still insist on circumcising their girls, prefer to do so in the pre-school years. In the traditional African viewpoint, uncircumcised girls are considered as delinquents, and those who avoid doing circumcision are faced with harsh reactions, sometimes accompanied with punishment in the tribe 50.

## Conclusion

During the last few decades, significant policies and actions in combating or reducing female genital circumcision were taken into effect in the countries of the Horn of Africa where these efforts are promising a better future for stopping this socio-cultural custom. As a joint report, by the UNICEF and United Nations Population Fund in 2018 indicates that due to the vast efforts of governments, NGOs, international organizations, and the support of the heads of the tribes and religious authorities, the number of female circumcisions in North-East African region is continuing to fall.

As a result, it is a possibility that in the coming decades, female circumcision is eventually recognized by the countries in the Horn of Africa as a traditional behavior that can be considered as a harmful practice. Yet, to eliminate this social contract from African societies, a strong platform is needed for change in the African ethnic and tribal behaviors and values. The researches carried out by international organizations suggest that the best remedy for the abolishment of female circumcision is close cooperation with the African people and local leaders. As a major authority for the elimination of female circumcision in the Horn of Africa, the World Health Organization believes that a total eradication would take a long time, possibly equal to three generations.^51^.

In fact, urban regions in the Horn of Africa are more culturally influenced by the educations of international organizations than those who live in outer villages, where there is a public distrust regarding the NGOs' movements, which causes obstacles in combating female circumcision. That is why the activists who are campaigning against female circumcision have clarified that they are not against the ancient ethnic tribal traditions of the African nations 8. Actually, the African elites have welcomed these efforts against female circumcision; nevertheless, the local villagers need a longer time to accept these efforts.

It can also be said that the number of female circumcision in the families with higher rates of education or those families that their daughter's education is important to them is very smaller. On the other hand, during the last two decades, the governments of these countries have included educational matters into school's curriculum, in addition, theses governments have begun cultural programs for teenagers and young urban and rural people about the girls' circumcision consequences and its future incurable physical and psychic results on their future. Moreover, training centers in the countries in the Horn of Africa have been trying in informing girls and women with their individual and social rights.

In the end, it is noteworthy that although there have been many articles on the Female Genital Mutilation in Africa, incuding the Horn of Africa, the author has attempted to have a new approach to this social phenomenon that has been a part of Africa's oral culture, passed down from one generation to the next. In fact what we can call as a new word in this paper is that the Horn of Africa is a highly varied region, made up of several ethnic groups, each with its own linguistic characters, norms, and customs. The Horn of Africa is comprised of highly patriarchal societies in which gender characters are well demarcated and great value is considered on females' premarital chastity and marital loyalty. So at-least in the Horn of Africa where FGM is wildly practiced, the practice is not regarded as a hazardous act or desecration of rights, but as an essential step to raise a girl ‘properly’, to guard her and to make her qualified for marital. Parents have to cut their daughters, therefore, to protect the best conceivable future for them. In fact, family dignity and social prospects play an influential role in preserving FGM; make it tremendously hard for individual families, to end the practice. Even after parents identify that FGM can cause severe damage, the practice keeps it up because they are afraid of negative repercussions and ethical judgments of society's expectations. The main encouraging force behind the practice is regularly the desire to safeguard and provide girls the best likely chance to have a future ensure them social acceptance and economic security in their own society. The wish to have girls married could be adequate to uphold FGM inside an assumed community. Though, social endorsement or condemnation, demonstrated over the community, also plays significant roles in continuing the practice. Disappointment to imitate FGM leads to social segregation, exclusion, displeasure, reproach or even ferocity – as well as having an outcome on a girl's marriageability. Therefore FGM in the Horn of Africa is a social norm and behavior that members of communities and ethnic groups have to follow it. In other word, although female circumcision has taken a different path than other ancestral traditions, it is still a part of that region's culture and any strategy to combat it should be done gradually and with caution.

Indeed, the most important message of this paper, which distinguishes it from other similar articles, is the necessity of deep respect for African traditions while at the same time promoting the explanation of the detrimental consequences of some customs that have to be either updated or set aside. Therefore, whilst respecting FGM as a deep-rooted custom, we have to convince and persuade the people in the Horn of Africa that the practice of female circumcision is harmful to the mental and physical health of those subject to it. At the same time, it is essential to show awareness that FGM is an ancient African tradition that to them, has to be respected. It should therefore be up to the holders of this tradition to gradually arrive at a place of self-judgment about it through their local leaders. This importance of this point was underscored through the exchange of information with several women in different Horn of Africa countries through interviews and questionnaires. Almost all participants emphasized that any action against FGM must be taken in a cautious manner and based on thorough knowledge of African customs, women's pre-existing assumptions especially in rural areas, and their gradual enlightenment via the existing framework of tribal chiefs. Most of the women interviewed pointed out that any struggle to eradicate FGM necessitates entering into an exceedingly sensitive and private arena, and needs patience, wisdom, and an understanding of African traditions directly connected to female destiny and identity. Perhaps for the first time, albeit anonymously, some African women expressed sincerely that whether their daughters want it or not, they intend to subject their daughters to female cutting in order to guarantee their futures and avoid the likely dishonor and disgrace that would encompass them should this not occur. This is very important because in most reported cases, African women have assigned the full responsibility of FGM to their husbands. But in this research—I reiterate again, on an anonymous basis—many women participants reported that they are not only actively participating in this practice but also (behind the scenes) intentionally encouraging their husbands to facilitate the mutilation of their daughters. This is the most important point of the present paper and a new and unique insight gained from the first-hand experience and knowledge of Horn of Africa women. In fact, most of the Horn of Africa women interviewed expressed a complex outlook on FMG: on the one hand, participants believe in female cutting and its continuation, although it is hard on their daughters, but at the same time, they want the practice to be stopped completely.

Therefore, the author's suggestion is that the fight against female circumcision requires enduring, consistent, and long-term planning, and the collective mobilization of statespersons, politicians, prominent cultural figures, artists, health centers, journalists, religious leaders, universities, educational institutions, and women's associations. It is also important to emphasize that any strategy geared toward combatting FGM in the Horn of Africa should avoid policies that may be perceived by the people in that region as aggressive and insensitive toward their traditions and cultural protocols.
